# What Psychiatry Means To Us

**DOI:** 10.4103/0973-1229.27613

**Published:** 2006

**Authors:** J. K. Trivedi, Dishanter Goel

**Affiliations:** * *Professor, Department of Psychiatry, King George's Medical University, Lucknow, India. E-mail: jktrivedi@hotmail.com, drjktrivedi@sify.com, drjktrivedi@sancharnet.in*; ** *Junior Resident, Department of Psychiatry, King George's Medical University, Lucknow, India. E-mail: dishanter@rediffmail.com*

**Keywords:** *Definition of Psychiatry*, *Psychiatry and Psychology*, *Scope and Field of Psychiatry*, *Diagnosis*, *Philosophical Basis*, *Good Psychiatric Practice*, *Ethics Professional Advancement and Research.*

## Abstract

Psychiatry has come up as one of the most dynamic branches of medicine in recent years. There are a lot of controversies regarding concepts, nosology, definitions and treatments in psychiatry, all of which are presently under a strict scanner. Differences are so many that even the meaning of psychiatry varies amongst individual psychiatrists. For us, it is an art to practice psychiatry and give the patient what he needs. Still, it should be practiced with great caution and utmost sincerity towards the patient, based on scientific knowledge and not to be guided by individual conceptions alone. Ethics in psychiatry forms an integral part of its basic concept and meaning, and a tight balance should be maintained between professional advancement and patient benefit. In recent years, the scope of psychiatry has enlarged considerably, with wide ranging influences from Sociology, Anthropology and Philosophy on the one hand, and Neurology and Medicine on the other.

## Introduction

To define the meaning of psychiatry is an intricate task. Each individual psychiatrist has his own perception of the branch. However, one thing that would be acknowledged by most of us would be that it is the source of our bread and butter. But would it be justified to recognize it merely as the source of our livelihood? Gone are the days when medical students chose psychiatry because they could not make their way to general medicine or surgery! Now most of them want to learn and practice psychiatry out of choice.

For us, it is a passion to practice psychiatry, a vast landscape waiting to be explored, a philosophy, a way to serve society and the needy, a method to decrease the sufferings of the psychologically disturbed, and benefit them in every possible way. It gives us great pleasure to talk to patients, make a diagnosis and treat them in accordance with the scientific knowledge available. Definitely, personal professional advancement is also a consideration, and we need make no bones about it. But that is never at the expense of patient welfare or scientific approach and practices.

## Defining Psychiatry

Discussion on the meaning of Psychiatry should definitely start with discussion over its definition. The term Psychiatry was first used by the German anatomist Johann Christian Reil (1759-1813; Campbell, 2004). Currian and Guttman (1949) defined “Psychiatry as a branch of medicine whose special province is the study, prevention and treatment of all types and degrees of mental ill health, however caused” (Slater and Roth, 2006). ‘Mental ill health’ is a vague term and undermines the importance of behavioral changes in a human, which might not necessarily qualify for being called ‘ill-health’. Slater and Roth describe psychiatry as “a branch of medicine in which psychological phenomena are important as causes, signs and symptoms, or as curative agents” (Slater and Roth, 2006). This definition takes into consideration only psychological phenomenon, which is retrogressive when considered in light of the conceptual advances in psychiatry made in the last few decades.

According to a recent definition by Campbell, Psychiatry is

The medical specialty concerned with the study, diagnosis, treatment, and prevention of behavior disorders (Campbell, 2004, p532).

This definition only considers the behavioral aspects of psychiatry. All psychiatric problems cannot be really expanded and applied to human behaviour as a whole, but in the wake of the current understanding of psychiatry, behavioral disorders should be included in the definition. We might go a little further and make a little change in Campbell's definition and say:

Psychiatry is the branch of medicine that deals with the causation, prevention, diagnosis and treatment of mental and behavioral disorders.

Though critics may argue that this definition does not specify the meaning of mental and behavioral disorders, they must realize that a definition must be precise and easy to understand, besides incorporating core features of the subject. We believe this definition best defines Psychiatry, in accordance with current knowledge and understanding in the field.

At present, we do not have the means by which many psychiatric illnesses could be cured completely. While some have short time courses and only minor symptoms, many are chronic conditions that can have a significant impact on patients′ quality of life, even life expectancy; and as such, require long-term or life-long treatment. Efficacy of treatment for any given condition is also variable from patient to patient. Some have complete resolution of symptoms while others unfortunately have poor or minimal response to even the strongest of measures. Majority of the patients fall somewhere in between.

In general, psychiatric treatments have improved significantly over the past several decades, beginning with the advent of modern psychiatric medications. In the past, psychiatric patients were often hospitalized for six months or more, with a significant number of cases involving hospitalization for many years. Today, most psychiatric patients are managed as outpatients. If hospitalization is required, the average hospital stay is around two to three weeks, with only a small number of cases involving long-term in-patient care.

## Contrast With Psychology

Psychiatry is practiced by psychiatrists who are medical doctors specializing in mental illnesses. They are trained in the medical approach to disorders and in the use of medications. Many (but not all) psychiatrists are also trained to conduct psychotherapy. Psychiatrists ideally evaluate patients from a biopsychosocial perspective before prescribing treatment. Biopsychosocial theories have evolved from the time of Alfred Russell Wallace, the man who, simultaneously with Darwin, put forward the theory of the evolution of species by natural selection. It has been rightly said:

*However great one's contempt for all theoretical thought, nevertheless one cannot bring two natural facts into relation with one another, or understand the connection existing between them, without theoretical thought. The only question is whether one's thinking is correct or not, and contempt of theory is evidently the most certain way to think naturalistically, and therefore incorrectly* (*Engels, 1878*).

Though the concepts have evolved greatly since his time, still the words of Freidrich Engels hold true in the evaluation of a patient by a psychiatrist, where theoretical knowledge of the subject becomes as important as the application of that knowledge on the psychological condition of the patient. This forms the basis of the biopsychosocial mode proposed by Engel (1982) as appropriate for all of medicine, wherein the psychiatrist evaluates the psychological and social aspects of a person in respect of the available biological knowledge. This is in contrast to a psychologist who does not give much consideration to the biological disturbances associated with psychiatric disorders.

Psychology is the larger study of human behaviour and thought processes. Psychology is as much an academic field of study (like biology or sociology) as a profession, and as a whole, is concerned with the study of normal everyday human behaviour as much as the study of mental illness. Psychologists may study how drugs or other chemical agents affect the brain, but generally are not trained to prescribe or administer drugs.

Clinical psychology is the branch of psychology that specializes in understanding and helping those experiencing mental distress or behavioral problems. Clinical psychologists have extensive postgraduate training in mental health, psychological assessment, psychotherapy, and psychosocial interventions, and are often found working in similar settings and with the same kinds of patients (or clients, as they prefer to call them) as psychiatrists. Unlike psychiatrists, they start with a general psychological training rather than a general medical training before going onto postgraduate courses. While psychiatrists may claim exclusive expertise in medication-based interventions and the general medical context, clinical psychologists may claim particular expertise in psychosocial interventions and the general psychosocial context, although the two are not always separated, or separable, in this manner.

Clinical psychologists are generally not authorised to prescribe medications. A significant subset of clinical psychologists argue that there is an inadequate number of psychiatrists for the number of people with mental health problems, and that focused education in psychopharmacology is adequate to provide medication management. We believe that clinical psychologists are currently not trained to prescribe drugs and thus they should not venture in this area. But, it should also not be forgotten that, psychiatrists are not always properly trained in psychological assessment and treatment. Rather it is always advocated that clinical psychologists and psychiatrists work in tandem and complement each other. As far as current knowledge regarding the understanding and treatment of psychiatric disorders goes, both of them are indispensable and neither the role of drugs nor that of psychological assessment and treatment can be undestimated.*

## Scope Of Psychiatry

Since man is a social animal in intimate association with his cultural environment, the level of organization, which should be studied, to provide an appropriate framework for psychiatry is that at which individual human beings are integrated with communities (Slater and Roth, 2006). There would be something to be said for this view if psychiatry could be justifiably defined as the ‘science of behaviour’. It would then have to aim at giving a complete account, not only of the clinical disorders that provide the daily work of most psychiatrists, but also of human motivation and behaviour as manifested in social and political life, in art and religion. This would include an expansion of the scope of psychiatry. A complete account of human behaviour in this sense would demand contributions not only from psychiatry, sociology, psychology and anthropology, but also from economics, history, literature and all sciences – in fact from all branches from human knowledge (Slater and Roth, 2006). The psychiatrist's knowledge should allow him to think about patients from the dual perspective of both biology and psychology in all clinical encounters (Gabbard and Kay, 2001). Since disordered behaviour touches upon many aspects of life in society, the psychiatrist is interested in knowledge obtained from the biological sciences (which contribute information on personality growth and pathology) and in scientific and humanitarian studies that contribute to the understanding of individuals with personality disturbances. These include Psychology, the Social Sciences, Law, Philosophy and Theology (Kolb and Brodie, 1982). Psychiatrists must have knowledge of normal developmental processes across the life cycle (physiological, psychological and social) and how these processes are manifested in behaviour and mental functions (Kolb and Brodie, 1982).

The IGDA 1994 project (IGDA means International Guidelines for Diagnostic Assessment, a collaborative effort between WHO and WPA) talks of assessment of the psychiatric patient as a whole person rather than just a carrier of diseases (IGDA, 2003a):

This assumes in the clinician the exercise of scientific competence, humanistic concern and ethical aspirations. Another essential feature is the coverage of all key areas of information (biological, psychological and social) pertinent to describing the patients′ disorders, dysfunctions and problems, as well as their positive aspects or assets (IGDA, 2003a).

## Field Of Psychiatry

The field of psychiatry itself can be divided into various subspecialties. These include:

- **Child and adolescent psychiatry** – this is the subspeciality of psychiatry dealing with mental problems of the child and adolescent age group. Over the years it has been felt that children and adolescents have a substantially different mental state than adults. Their demands and behaviour, and its determinants, are different and the presentation of their illnesses is also different. In fact many disorders have now been identified which are exclusive to this population. Likewise, the treatment differs in terms of the choice of medications and their dosages. So a need for a special training of a psychiatrist in child and adolescent psychiatry was long felt. Unfortunately it has still not attained the same recognition in India as in the West, but is growing at a rapid rate.
- **Adult psychiatry** – this is general psychiatry, which includes basic training in the branch, and is what is commonly known and practiced as psychiatry.
- **Old-age psychiatry (Geriatric psychiatry)** – this is a recent discipline of psychiatry which has found its way due to the realisation that, like children, the elderly also have different pharmacokinetics of drugs, different presentations of disorders, and also disorders exclusively seen in this age group. The needs of the elderly population are different from those of the adult age group and this needs to be addressed in a more specialized way. Supporting this view the first department of Geriatric mental health in India has come up at K.G.Medical University, Lucknow, in August 2005.
- **Sexology** – sexology has been striving hard to segregate from psychiatry. But till date sexology is only taught under the purview of psychiatry in most medical colleges in India. It is currently not so advanced as a medical science in India as to be studied as an altogether separate speciality, and it would be immature to carry out any segregation at present.
- **Consultation-liaison psychiatry** – most of the times undergraduate training in psychiatry is inadequate. So general physicians, surgeons and other specialists find it difficult to identify psychiatric manifestations of physical disorders or comorbid psychiatric disorders. Thus, it becomes extremely important for them to have a psychiatrist with their expert team and, based on this view, consultation-liaison psychiatry has been growing recently.
- **Emergency psychiatry** – psychiatric emergencies can be disruptive not only for the patient but also for society, family or for national property. Inadequately trained psychiatrists often find it difficult to diagnose or deal with emergencies. Emergency psychiatry is gaining popularity in western countries but still has a long way to go in India.
- **Addiction and substance abuse psychiatry** – it is always challenging to deal with substance abuse related problems and emphasis on rigorous training in this subspeciality is being given at various centers in India*.
- **Forensic psychiatry** – as psychiatric patients are more prone to abuse, it becomes essential to have a complete knowledge of laws of the state as related to a mentally challenged person. Usually, during general psychiatry training much emphasis is not given to legal implications in and of psychiatry. Hence it becomes necessary to give specialized training in forensic psychiatry to the willing psychiatrist.

It is desirable that psychiatrists attempt to define the social and environmental conditions under which the individual thrives or breaks down. The psychiatrists must play his part in defining those environmental, domestic, occupational, economic, habitual and nutritional factors without which the intimate causal factors cannot find their opportunity. But he must retain a sense of proportion, and, in practical work as in research, direct his main energies to those tasks in the field where needs are most pressing, development most promising and where his special skills and experience are most likely to make him effective. For, if he should dispense his activities over too wide a field, he will inevitably fall a victim to superficiality and error (Slater and Roth, 2006).

## Making A Diagnosis

Many psychiatrists argue that psychiatry lacks disease specific pharmacological approaches. Thus, for prescribing medications it is not necessary to intensively classify illnesses. Rather, it can be very well simplified by just making sure whether it is a schizophrenia spectrum illness or a mood disorder or anxiety disorder.

But this is a hazardous way of thinking. If we forego the making of a diagnosis, we also forego all applications of the extensive knowledge that has been accumulated in the past. This would be sheer folly. We cannot willfully ignore what is known. The wise psychiatrist never neglects the individual peculiarities of his patient; but he will first see how far he can be fitted into general patterns and he will not neglect a quality that is characteristic of the group. Hence both individual peculiarities and a standardized diagnostic formulation are needed.

The IGDA (2003b), while discussing its conceptual basis, emphasizes the nomothetic - idiographic integration an essential, and makes the following observation:

The diagnostic process involves more than identifying a disorder. Positive aspects of health, such as personal and social assets and quality of life, should also be described. The diagnosis itself should combine a nomothetic or standardised diagnostic formulation (e.g. ICD-10, DSMIV) with an idiographic (personalised) diagnostic formulation, reflecting the uniqueness of the patient's personal experience. At the nomothetic level, a multi-axial diagnostic formulation is recommended. For the idiographic formulation an integration of the perspectives of the clinician, patient and family should be presented in natural language (IGDA, 2003b).

Commenting on the nomothetic-idiographic integration, Singh and Singh (2004) say:

In other words, the standardised must gel with the personalised and the socialised to make for good psychiatric practice. It is a moot point whether a nomothetic-idiographic orientation is valid only for psychiatry, or for all of medicine (Singh and Singh, 2004a).

Classification helps us to determine the prognosis, course, and decision to use the most appropriate drug from a particular group. If these classifications are never made, naturally, the information they provide never accrues. Furthermore, if we are going to allow ourselves only one method of treatment e.g. pharmacotherapy or psychotherapy, and apply it to every case, no information of a general kind can possibly be relevant to treatment. Rather, such an approach takes us back to the days of universal purging and bleeding.

Diagnosis is not a matter of merely naming and labelling. Ideally it implies judgement of causation and also includes a plan of action. Even while making a diagnosis, interpretation and understanding of clinical phenomenon is of prime importance.

## Differences In ‘What Is Thought’ And ‘What Is Taught’ In Psychiatry

Although practicing psychiatrists share much in common, there is rather too much dissension in Psychiatry at present to be regarded as entirely healthy. Psychiatry is one of the branches of medicine that faces the brunt of being guided by individual concepts rather than population based researches. Wide differences about fundamental issues exist between what is thought and taught at different centres. These differences inspire attitudes of dogmatism, and there is not the open mindedness there should be. Rapid advances are being made but are judged or even ignored on the basis of preconceptions. Theoretical exposition follows theoretical exposition in ever-growing complexity and the need constantly to check theory by seeking at every point for new evidence is forgotten. The solid acquisitions of knowledge from the past, where they conflict with current modes of thought, are not being reformulated where necessary, but are being neglected and even forgotten. Psychiatry is not only being split into a number of schools but is also divorced from the parent science of medicine.

Growth in every field, in the number of practicing psychiatrists, in the amount of time given to psychiatric teaching of undergraduate and postgraduate students, in the claims made by psychiatrists to be heard in their own and related fields, in public esteem and support, has led to a corresponding decrease of discrepancy in the practice and literature in psychiatry. Various conferences, CMEs and other academic gatherings at the national and international level serve to bring uniformity in psychiatric thinking and also to bring it more close to the available literature.

## Philosophical Basis Of Psychiatry

Over the years, in the wake of new findings and conceptual advances, psychiatry has landed in a peculiar position, between medicine and neurology on one side and philosophy and psychology on the other.

It would be prudent to remember that, as recently as 100 years ago, the treatment of mental aberrations was regarded as the province of philosophers or theologians. Since long, many have tried to solve the problems of psychopathology by the use of philosophical short cuts, instead of the relatively slow method of investigation with the disciplines of the natural sciences. Due to its nature, existential analysis appeals to those of a philosophical or metaphysical bent of mind. Lacan (1977) brings psychoanalytic theory into close contact with the philosophy of mind and psychiatry as illuminated by the continental tradition. He draws on Freud, phenomenology, existentialism, and structuralism to construct a subtle theoretical approach to the psyche according to which our engagement in discourse and our existence in the world combine to generate a many-layered structure of meanings and influences that form us. This allows him to focus on the nature and role of language and our discursively constructed self-conceptions both in the therapeutic encounter and in the maladies of the psyche. The relevance of Lacan's thought for analytic philosophy of psychiatry can be explored by pursuing his links with Freud, his complex treatment of issues in the philosophy of language, and a subtle blend of existentialism and continentally inspired naturalism that paints the contours of the unconscious mind in operationally compelling terms (Lacan, 1977). Freud draws on phenomenology and evolutionary theory to argue that even though the psyche must be understood in its own terms, human neurobiology is its basis (Freud, 1986). In fact psychiatry can never be segregated from the basic philosophy of life as concepts like religion, culture, stress, loneliness, sleep, dreams, politics will always play their influence in the human mind and vice versa.

Religion and Psychiatry are not in conflict: both enjoy an altogether different perspective and the role of religion cannot be undermined, even from a psychiatrist's point of view.

Neither scientific temper nor religious beliefs are complete methods in themselves to explain all phenomena. Scientific temper gives supremacy to evidence and reason; religious belief gives supremacy to introspection and intuitive experiences. For holistic understanding of phenomena, both approaches are necessary. A healthy interaction between them, and their fusion, is necessary both at the social and the individual level. They are not only competing but complementary approaches (Singh and Singh, 2004b).

As has been so aptly said by Albert Einstein:

Science without religion is lame, religion without science is blind.

Theologians argue that the cure of the psyche is helpful to, but no substitute for, the cure of the soul (Thevathasan, 2006). Religion has always been one of the key determinants of human behaviour and thus it can never be separated from psychiatry, nor can its influence on the presentation of psychiatric disorders be discounted. Psychotherapy and the greater part of western religious thinking, however, share a belief in the existence of a transcendent mind. Recent developments in cognitive science and certain spiritual traditions challenge this implicit mind-body split, providing an opportunity for a renewed dialogue between psychiatry and religion and the possibility of collaborative research (Bathgate, 2003). An epistemological gap most certainly exists, but there is a growing acceptance of the importance of religion and spirituality to psychiatry. Rapprochement may best be achieved by increasing psychiatric awareness and knowledge of the issues, and by a willingness to embrace intellectual, cultural and religious pluralism (Turbott, 2004). Likewise culture has been one of the major influences on human behaviour. So much is the influence of culture that specific culture bound syndromes do occur in a person.

## Essence Of Good Psychiatric Practice

A good psychiatric practice is always based on scientific evidence rather than being guided by individual preferences and clinical experiences alone. Individual clinical experiences are often misleading and management plan should always be supported by planned research data. Thus a good psychiatrist should have comprehensive knowledge of the subject and should also keep himself updated for new advancements in the field. With regard to many phenomena that he has not still studied, he should, like a true scientist, withhold comment:

The true scientist withholds himself from passing comment on a phenomenon which his experimental method can either not verify or which falls outside the purview of his branch itself. That does not mean he shirks his responsibility. It does not also mean he may not take up this phenomenon for study at a future date, when he develops the necessary methodology and the expertise (Singh and Singh, 2004b, p 64; 2005).

Another important aspect in the practice of psychiatry is the satisfaction of the patient and his family. Most of the times, the patients are not very demanding. All they want is explanation of their disease and treatment, which is not a big deal to ask for. A good psychiatrist should be supportive and respectful towards his patients and the family.

## Ethics In Psychiatric Practice

It is our observation that it is easier to be a rich psychiatrist but more difficult to be a good psychiatrist. But a good and ethical psychiatrist is usually a rich one too.

Often psychiatric patients are prone to abuse - sexual, physical and financial, and their families get distressed. If, in such conditions, we charge them heavy fees, ask them to get undue investigations done, and overburden their pocket by prescribing costly drugs, we are adding to their sufferings rather than helping them in any way. Can we call ourselves doctors in that case? Can we call ourselves humans? Well, it sounds too ‘philosophical’ to think of, but the fact is that this philosophy should accompany us from the day we step into our medical course and stay there for the rest of our life. This is especially important in our country where patients are often less resourceful, and insurance cover for most psychiatric illnesses non-existent

Ethical practice is the real meaning of psychiatry to us. It is, in fact the crux of good psychiatric practice.

Ethics further encompasses activities like helping fellow practitioners whenever needed, referring the patient to the proper specialist, and avoiding trying one's hands in areas beyond one's expertise.

## Professional Advancement In Psychiatry

Many practitioners refute/sideline ethics, citing the need for professional and monetary advancement. But psychiatrists who are more ethical in their practice go on to achieve greater success in life and they are able to enjoy their success more than their unethical counterparts. As has been rightly said:

While success is important, it can become enduring only if it is based on a strong foundation of values. Define what you stand for as early as possible, and do not compromise with it for any reason. You can't enjoy the fruits of success if you have to argue with your own conscience (Premji, 2004).

The face of psychiatry has changed dramatically in the past fifteen to twenty years. In our country, hardly thirty years back, whoever took up psychiatry was considered a failure, a man unable to make his way in medicine or surgery. This has now changed and the psychiatrist is recognized as a physician of standing as well as a counsellor on social problems of general interest. Even private practice is legitimate, as well as private enterprise in psychiatry (as in all medicine), provided patient welfare is never compromised:

*It is important that the profession of medicine should accept and allow for private enterprise in the field if it does not want nefarious activities to prosper clandestinely. Something like what it did for abortion. Not allowing it caused so much illegal trafficking, allowing it with safeguards took the sting out of malevolence. Something similar would happen if we accept the enterprise of medicine as legitimate, but with the necessary condition of patient welfare not to be violated at any time. That proportion of patient welfare which ensures profit is necessary. And that proportion of profits which never neglects patient welfare is as necessary. For, to try to ensure patient welfare without profits is a lame exercise, and would always fail. Just as to ensure profits without patient welfare is a blind enterprise, and would always falter. So, if we do not wish to fail, or falter, the only resolution is that proportion of patient welfare which also ensures profits, and that proportion of profits which also ensures patient welfare. Any compromise in this formulation and we know what it means (Singh and Singh, 2005-2006)*.

## Research In Psychiatry

Progress depends on recognizing similarities in phenomena that may superficially differ greatly, for, from these recognized similarities we may deduce general causes. Any single and simple hypothesis can be regarded with suspicion from the start. This does not mean a theory be rejected offhand. It should, rather, be carefully tested and its implications explored; for it may provide a useful framework for a limited range of data, suggest fruitful subjects for research and, if substantiated, eventually prove capable of organization into a larger scheme. In order that science progresses, the mind needs working hypotheses in order to grasp and dissect experiences; and no harm is done if these hypotheses are only partial, or even faulty, provided they are invariably regarded with skepticism, accepted only provisionally as long as evidence supports them, and refuted when fresh evidence compels us to do so. This is the essence of scientific progress. For:

Anything that is not objectively verifiable, that cannot be experimentally proved and does not have the possibility of replication cannot fall within the purview of scientific investigation or research (Singh and Singh, 2004b, p 61; 2005).

Psychiatry has come up as one of the most dynamically changing branches of medicine in recent years. So much is the influence of research in psychiatry that it just cannot be segregated from the basic meaning of psychiatry nowadays. The focus of research has now shifted from psychological influences to the biological basis of mental illnesses. Whenever a new branch of scientific activity appears with some relevance to human behaviour, or whenever a new concept meets a popular response, psychiatrists seize upon it and try out its implications in their own field. This also is a way of breaking new ground which must, however, be worked over by the methods of science if it is to bear fruit.

## Concluding Remarks

- Understanding and defining the meaning of psychiatry is a difficult job, not easy to put into words.
- Even after so many years of its inception, understanding psychiatry has been an enigma.
- It has a complex relationship with religion, culture, philosophy and science, which is not properly understood till date, and opinions vary widely.
- Even more enigmatic has been the human brain, which is the biological substrate for emotions, cognitive abilities and behaviour - that is, everything that humans feel, think and do.
- A useful way of looking at it is to accept the statement below:

A paradox in the understanding of the human mind is that it is wise to know everything there is to know, but nothing is to be believed (Sadock and Sadock, 2004).

### Take Home Message

- In recent years, growth of psychiatry has served to reduce self-criticism by psychiatrists all over the world and has also infused more belief in its capabilities. More research, thorough introspection and an enhanced understanding of the subject can earn greater self-belief for the branch.
- This could probably take us to the era of *evidence based conviction* in psychiatric concepts rather than just knowing, or believing in, them.
- Clinical psychiatric practice should always be ethical, even if consideration of professional advancement is not discounted.

## Questions That This Paper Raises

Q.1 How do we define psychiatry?
Q.2 If the psychological aspect is dealt with by psychology and the biological aspect by neurology, what is the need for a middle path, psychiatry, between the two?
Q.3 If clinical psychologists are trained in pharmacotherapy, will it mark an end of psychiatry?
What all is to be included under psychiatry – what is its defining scope?
Q.4 Do philosophical concepts still find legitimate place in psychiatry?
Q.5 Why shouldn't psychiatry be dealt with as a completely biological science, when the biological basis of most of psychiatric disorders have been found out?

## About the Authors

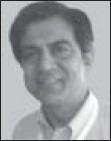



***Professor J.K.Trivedi*** *(Principal and Corresponding Author) is a sound clinician, researcher, has an in-depth knowledge of psychiatry and is currently Professor in the Department of Psychiatry, K. G. Medical University, Lucknow. He is a renowned teacher and academician having made presentations on various aspects of psychiatry both nationally and internationally. He has been Editor of Indian Journal of Psychiatry (IJP) for six years and has been associated with IJP for more than 18 years in various capacities. He was President of Indian Psychiatric Society for the year 2004. Under his dynamic leadership the IPS has flourished to greater heights. He is currently Zonal Representative for Southern Asia - Zone-XVI of World Psychiatric Association. He is also on the International Advisory Board of the Mens Sana Monographs (MSM).*

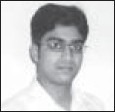



***Dr. Dishanter Goel (Co-Author)*** *is currently pursuing his master's degree in Psychiatry, from Department of Psychiatry, K. G. Medical University, Lucknow. He has completed M.B.B.S. from K. G. Medical University, Lucknow and has been a meritorious student throughout. He is academically and clinically oriented and has been an active participant in various national and zonal conferences. During his tenure of residency, he has got training in the fields of general adult, child and adolescent, geriatric, sex clinic and de-addiction psychiatry.*

## Footnotes

* Sea also Editorial, Psychiatrists And Clinical Psychologists, pg10-13. Also see pg3649 and pg139-153 (eds).

* See also pg104-126 (eds).
